# Towards a Universal Approach Based on Omics Technologies for the Quality Control of Food

**DOI:** 10.1155/2015/365794

**Published:** 2015-12-13

**Authors:** Emanuele Ferri, Andrea Galimberti, Maurizio Casiraghi, Cristina Airoldi, Carlotta Ciaramelli, Alessandro Palmioli, Valerio Mezzasalma, Ilaria Bruni, Massimo Labra

**Affiliations:** ^1^FEM2 Ambiente s.r.l., P.za della Scienza 2, 20126 Milan, Italy; ^2^ZooPlantLab, Department of Biotechnology and Biosciences, University of Milano-Bicocca, P.za della Scienza 2, 20126 Milan, Italy; ^3^BioNMR Lab, Department of Biotechnology and Biosciences, University of Milano-Bicocca, P.za della Scienza 2, 20126 Milan, Italy

## Abstract

In the last decades, food science has greatly developed, turning from the consideration of food as mere source of energy to a growing awareness on its importance for health and particularly in reducing the risk of diseases. Such vision led to an increasing attention towards the origin and quality of raw materials as well as their derived food products. The continuous advance in molecular biology allowed setting up efficient and universal omics tools to unequivocally identify the origin of food items and their traceability. In this review, we considered the application of a genomics approach known as DNA barcoding in characterizing the composition of foodstuffs and its traceability along the food supply chain. Moreover, metabolomics analytical strategies based on Nuclear Magnetic Resonance (NMR) and Mass Spectroscopy (MS) were discussed as they also work well in evaluating food quality. The combination of both approaches allows us to define a sort of molecular labelling of food that is easily understandable by the operators involved in the food sector: producers, distributors, and consumers. Current technologies based on digital information systems such as web platforms and smartphone apps can facilitate the adoption of such molecular labelling.

## 1. The Demand for Universal Analytical Tools to Characterize Foodstuffs

The globalization of the food market has led to a corresponding increase in issues concerning the authenticity and safety of imported foods. Consumers are susceptible to any form of food alteration that may occur during artisanal or industrial manufacturing processes and pay attention to food ingredients as these can influence nutritional and health conditions [1–3]. The consumer's awareness in terms of food quality and safety is growing and growing and implies the search for products with exhaustive labelling reporting details about the original raw materials and with assurances about the absence of harmful chemical and microbial contaminants [4–6]. These topics drove the development of new analytical tools in the context of food science [7]. A relevant section of approaches was the one devoted to the screening of undesired microorganisms, often occurring in foodstuffs, to ensure human safety and preventing food spoilage and/or the spread of foodborne disease outbreaks [8, 9]. Foodborne pathogens, as well as spoilage microorganisms, can already be present in the indigenous microbiota of raw materials or colonize the final food product by contamination during manufacturing [10]; therefore, laboratory analyses must be conducted both on raw materials and transformed food items. There is a great number of microorganism taxa traditionally associated with human diseases and for which every food product should be tested in order to ensure their absence.* Salmonella* spp. is one of the major pathogens responsible for foodborne disease outbreaks throughout the world and* S. enterica* is the most frequently isolated species [11]. Other important and frequently reported foodborne pathogens belong to the genera* Campylobacter*,* Yersinia*,* Shigella*,* Vibrio*,* Clostridium*,* Bacillus*,* Listeria*, and* Staphylococcus* [12, 13]. Most of these microorganisms are not easily detectable with culture-dependent approaches, but DNA-based tests that improve their detection have been developed. Most of these are based on the simultaneous detection of a wide panel of entities by using universal DNA marker regions such as the 16s rDNA or the ITS [14, 15].

DNA-based approaches have acquired a growing importance also to respond to another consumer's request that is the authentication of both raw materials and processed food products [1]. Such a demand arose due to different factors: (i) the globalization of the food market that caused a longer and more articulated food supply chain, where raw materials are globally exported and processed in countries different from the origin; (ii) the industrialization of manufacturing processes (e.g., fermentation, biopreservation, and functionalization [16]) that are becoming more and more complex and largely unknown to the consumers; (iii) the strong modifications to which foodstuffs are subject before being sold (e.g., slicing and powdering) that impede a correct identification of the original raw materials by the consumer; (iv) the growing occurrence of allergies and intolerances related to certain foods or components of processed foodstuffs, typical of western countries. A plethora of molecular-based tools has been developed to characterize food composition and validate food authenticity [1], most of which relying on the analysis of proteins [17] and/or DNA sequences [18]. Protein-based approaches are useful in characterizing the composition of fresh products; however, these methods can be biased by several factors such as the strong food manufacturing processes, the limited number of detectable isozymes, or the high tissue and developmental stage specificity of the markers [19]. DNA markers were definitely proven to be more informative than protein-based methods because DNA better resists industrial processes such as shredding, boiling, pressure cooking, or transformations mediated by chemical agents [20, 21]. This property allows a successful identification of animal, plant, or fungi raw materials, even when they are present at small traces. Moreover, the availability of advanced technologies and efficient commercial kits for DNA extraction permits obtaining an acceptable yield of genetic material from processed or degraded biological material [8, 22, 23].

DNA analyses in food science are based on specific genome regions used as “identity markers” easily detectable by Polymerase Chain Reaction (PCR) [18]. Discontinuous molecular markers such as Amplified fragment Length Polymorphisms (AFLPs), as well as their variants (i.e., ISSR, SSAP, and SAMPL), have been successfully used in the characterization of several food raw materials [18, 24]. Moreover, species-specific makers have been developed for the most important and traded categories of animal and plant raw materials. This is the case of Single Nucleotide Polymorphisms (SNPs) and Simple Sequence Repeats (SSRs) that are largely used because of their high level of polymorphism and high reproducibility [25]. These approaches are used both in the identification of plant cultivars [26, 27] and animal breeds [28, 29] and to prevent fraudulent commercial activities [30, 31]. However, being highly species-specific, these approaches require a deep knowledge of the genotypes of the organisms and their application is often limited to a single taxon, or to a few closely related taxa. Nowadays, producers, manufacturers, distributors, and consumers advocate the development and adoption of universal tools to assess not only the origin and traceability of raw materials and derived food products but also the inadvertent occurrence of other species (i.e., contamination) or cases of species substitution (i.e., frauds). The development of innovative food-related universal tools based on DNA analysis will be the first issue treated in this paper.

However, the DNA certification of identity and origin of foodstuffs are not necessarily synonyms of food quality. As an example, the genetic identity of a vineyard influences some aspects of wine quality [32] but other environmental factors could affect the plant phenotype and therefore the wine organoleptic properties [33–35]. For these reasons, the DNA-based analysis should be combined with a precise evaluation of chemical food characteristics. The second section of this paper will be devoted to the analysis of modern metabolomics techniques in the field of food science.

Both DNA-based and metabolomics approaches can be simultaneously performed through the so called omics platforms [36], the use of which is expected to progressively become a routine in the context of food control. Given the recent bioinformatics advances, omics platforms are able to process huge amounts of data and combine information belonging to different analytical approaches. Hence, the technological innovations concerning food quality lie in both the development of universal and more accurate analytical systems and their reciprocal integration.

## 2. DNA Barcoding: A Universal Approach for Food Characterization

As discussed in the previous chapter, an aspect of primary importance in food science is the need to identify the origin of food raw materials, as well as tracing food products along the entire food supply chain by using universal, rapid, and inexpensive tools. In the last decade, “DNA barcoding” was proposed as a universal method to identify living organism including edible plants and animals [37]. The rationale of this approach consists in the analysis of the variability at one or a few standard region/s of the genome (i.e., DNA barcodes) occurring in the whole panel of organisms constituting the raw materials and their derived food products [38].

The 5′-end portion of mitochondrial* coxI* gene was suggested as standard DNA barcode region for metazoans. In plants, mitochondrial DNA has slower substitution rates and shows intramolecular recombination [39], therefore impeding a reliable species identification. The research for an ideal DNA barcode in terrestrial plants has focused on two plastid DNA regions (i.e.,* rbcL* and* matK*) considered as the “core-barcode” [40]. These can be supported by other regions, such as the* trnH-psbA* intergenic spacer, due to their higher variability among congenerics [41, 42]. Internal transcribed spacer regions of nuclear ribosomal DNA (ITS) were also recommended as additional markers in angiosperms [39].

Although there is still much debate on the identification performances of these markers, DNA barcoding showed its effectiveness when used to characterize unknown specimens based on the comparison with reference sequences [42, 43], especially for edible organisms used in food production [44–47]. The efficacy of DNA barcoding is supported by the availability of a comprehensive and continuously growing public library of DNA barcodes, the Barcode of Life Data System (BOLD), which provides a global identification system that is freely accessible [48, 49]. This platform consists of several components, including the Identification Engine tool (BOLD-IDS), which works with DNA barcode sequences and returns a taxonomic assignment at the species level whenever possible.

A case in which DNA barcoding works well is the analysis of seafood [50], where* coxI* showed higher discrimination ability and in several cases allowed the identification of the origin of certain fish stocks. Moreover, in the modern market, many seafood species are sold as fillets or slices, therefore hindering the application of classical identification approaches. In such cases, the molecular analysis is the only reliable strategy to identify species [51]. Given its efficacy, DNA barcoding was adopted by the US Food and Drug Administration for the authentication of fish-based commercial products [52].

A limited success of the method was achieved concerning meat identification, especially concerning farmed species. The main reason of this pitfall lies in the scarce variability of the conventional barcode region among animal breeds and in the frequent occurrence of hybridization events [53]. In contrast, regarding dairy products, DNA barcoding has been proven efficient in characterizing composition and origin of milk. Indeed, the plastidial* rbcL* barcode marker was found to be able to detect traces of food-derived plant DNA fragments in raw cow milk [54, 55], thus opening new perspectives for the traceability of milk and dairy products in general.

Among plant-based foodstuffs, the DNA barcoding approach has been used for many applications [56] and to investigate the genetic relationships between wild and cultivated plants, as well as their origin. As an example, DNA barcoding was used to characterize the bean germplasm (*Phaseolus vulgaris* L.) and was found able to distinguish among different haplotypes of bean accessions from the Mesoamerican and Andean areas [57]. Similarly, the DNA barcoding approach was adopted to assess the origin and quality of spices [44, 58], herbal products [59, 60], and naturally processed plant products such as multiflower honey [61]. Other studies investigated the ability of DNA barcoding in discerning toxic plants from edible species: cultivated species of the genera* Solanum* and* Prunus* were successfully distinguished from their toxic congenerics [62] and from some frequent plant species misidentifications that cause poisoning in human [63].

On the whole, the most important innovation introduced by DNA barcoding is the merging in a single approach of three characteristics typical of molecular analytic tools: (i) the* molecularization* of identification processes (i.e., the investigation of DNA variability to discriminate among taxa); (ii) the* standardization* of molecular marker/s and of analytical procedures; (iii) the* data computerization* of identification results (i.e., the not redundant transposition of the data using informatics) [64]. This last element is fundamental to make the analytic DNA-based tool accessible to the different actors involved in the food supply chain.  Table 1 provides an updated list of DNA barcoding case studies dealing with raw materials and foodstuffs with a clear indication of the beneficiary subjects of the analysis: producer, distributor, and consumer.

Although DNA barcoding largely demonstrated its high sensitivity and reliability in the authentication of food products, it should be specified that most food products are composed of a mix of organisms. In these cases, the use of universal primers and standard sequencing approaches, based on the traditional Sanger technology, are inefficient to discriminate among the single components. As a result, the requirement for high-throughput sequencing techniques grew by an unpredicted extent [106]. Several novel approaches evolved to replace the traditional Sanger sequencing method; these modern advances have been referred to as “high-throughput DNA sequencing” (HTS). HTS techniques are able to provide billion sequence data several times faster and cheaper than the conventional Sanger approach. The reduction in cost and time for generating DNA sequence data has resulted in a range of new successful applications, including food traceability and especially food microbiology [16, 107]. As an example, HTS techniques have been used to identify fruit species in yogurts [108] and pollen composition in multiflower honeys [109].

Nowadays, the use of DNA barcoding in the food sector moved from the academic research to a real application. The “molecular labelling” provided by DNA barcoding has benefits for both consumers (who are ensured on the origin, quality, and safety of food items) and producers (who can give an additional value to their products or have an assurance on the quality of starting raw materials). Concerning the analytical feasibility of the method, the DNA barcoding tool is easily accessible due to the availability of public molecular reference databases and a lot of equipped public or private laboratories able to perform the analysis. Newmaster and colleagues, in a publication dated 2009, estimated the cost of a single analysis in a few Euro and very short times of response [110]. Federici and colleagues demonstrated that portions of the standard DNA barcodes could be chosen as SCAR markers to discriminate in less than three hours between edible plant species from poisonous ones [63]. These characteristics make DNA barcoding a diagnostic method suitable for food control analyses by national and international agencies. As previously underlined, to assess the origin of food items, DNA-based analyses should be combined with the characterization of food metabolites to obtain an exhaustive molecular label.

## 3. Innovative Applications of Metabolomics Tools for an Exhaustive Food Labelling

The analysis of food metabolome represents a new frontier in the evaluation of food quality [111]. The metabolome consists of low molecular weight entities (i.e., <1,000 Da) [112] belonging to a wide range of chemical classes, occurring at different concentrations. In general, these metabolites are the final downstream products of the genome and of its interactions with the environment. For this reason, the analysis of genotype only (e.g., DNA barcoding) is certainly important but not exhaustive to evaluate the overall quality of food items.

In food chemistry, some molecules such as sugars are common and abundant, whereas minor compounds like vitamins occur at smaller amounts or even at trace concentrations (e.g., femtomolar). In addition, the physicochemical properties of some groups of molecules, or the patterns of reciprocal interaction, could pose problems to their fine characterization and quantification. Thus, efficient and sensitive analytical tools are required for a reliable characterization of food metabolome. Whilst in DNA fingerprinting approaches the identification is based on the reading of short nucleotide DNA sequences, a metabolomics fingerprinting analysis aims at establishing the patterns of metabolites belonging to different chemical classes and that are correlated to certain characteristics. Thus, one of the main challenges in food metabolomics is facing the complex networks of molecules (e.g., sugars, amino acids, peptides, organic acids, phenols, terpenes, or steroids) occurring in a particular food item. For these reasons, two approaches (*profiling* and* fingerprinting*) can be used to characterize the food metabolome. Profiling is a targeted strategy focused on the analysis of a group of related metabolites, often belonging to the same chemical class. An example of this approach is the discrimination between Arabica and Robusta coffee origins, based on the identification and quantification of a specific class of molecules, including 16-*O*-Methylcafestol, by NMR spectroscopy [113]. In addition, very recently, Monti and coworkers discriminated among different peach qualities and level of ripening, which depend on the abundance of several metabolites, including amino acids, sugars, and organic acids [114]. The second approach (fingerprinting), is an untargeted strategy based on comparing patterns of metabolites among different samples using chemometric tools. The main aim of fingerprinting is not to identify all the involved compounds but to establish patterns among them; this approach enables the simultaneous detection of a wide class of metabolites. Examples of metabolic fingerprinting on different foodstuffs include grape and wine [115, 116], orange [117], saffron [118], olive oil [119], and wheat and bread [120]. Profiling and fingerprinting can offer complementary information and thus can be used alone or in combination [121, 122].

Independently from the adopted strategy, a reliable tool to analyse the metabolome of a certain food should ideally meet some features: (i) the possibility of recognizing a variety of chemical structures, (ii) the possibility of dealing with large range of concentrations at which metabolites are present in a matrix, (iii) the capability of the analytical platforms, and (iv) the availability of reference databases with extensive details and descriptors [123].

**Figure 1:**
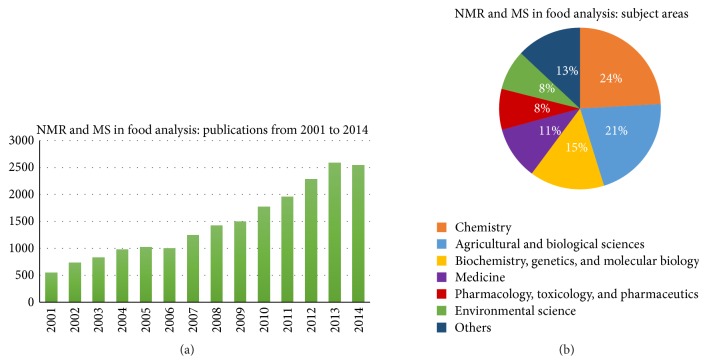
(a) Studies published in the area of food research, based on NMR and/or MS analyses, from 2001 to 2014. (b) NMR- and/or MS-based studies published from 2001 to 2014, divided for subject area. Source: Scopus (entries: NMR, food or Mass Spectrometry, food).

Today, there are two analytical platforms meeting these criteria: Nuclear Magnetic Resonance (NMR) spectroscopy and Mass Spectrometry (MS) [121]. The application of NMR and MS techniques greatly increased in the last years (Figure 1(a)) and this research field covers several subject areas and disciplines (Figure 1(b)).

A good advantage of both techniques is the “high-throughput” capability of spectroscopic and structural information that permits characterizing a wide range of metabolites simultaneously, with high analytical precision. Compared to NMR, MS is more sensitive and can be used alone or combined with gas chromatography, liquid chromatography, or capillary electrophoresis to provide a higher sensitivity for metabolites present at low or even at trace concentrations [124–127]. However, even though MS-based analytical methods can detect hundreds of metabolites, many others could remain unidentified. On the other side, the main advantages of NMR are the ease sample preparation and the determination of very different chemical species in a single experiment. In addition, the identification of molecules is easier and more straightforward than in the case of MS. Other important advantages of NMR are its inherently quantitative signals and its nontargeted and nondestructive nature with regard to the specimen of the technique. Thus, in case of an initial metabolomics study where the composition of the metabolite pool is not known, a NMR approach is useful and can inform future studies by targeted GC-MS metabolomics or other approaches to look for specific low-concentration metabolites (targeted strategy). NMR sensitivity is considered one of the main limitations in its application to metabolomics analysis, especially when compared to MS. However, continuous developments in hardware (e.g., magnet strength, probe head design, and console electronics) have allowed and will allow a growing sensitivity of NMR. Also, a rapid growth in new, potent algorithms for multivariate data analysis facilitates the use of NMR spectroscopy as a competitive, complementary analytical platform for investigating the food metabolome (Table 2).

The most important innovation provided by metabolomics tools is their standardization and the universality of the procedures. The amount of data generated by these analyses is enormous. For this reason, several chemometric tools [139, 140] are employed. In fact, to analyze food metabolomics data, some intermediate steps are necessary, including peak detection, spectra normalization, integration, and data alignment before multivariate statistical analysis.

Based on these aspects, it is currently possible to create a molecular label, which combines the genetic profile of a certain food item and its metabolic content. The advantages of such integration are relevant and would certainly constitute a real innovation in food science. One example is the case of wine, which can be putatively characterized with both DNA analysis of the original grape cultivar (e.g., [141, 142]) and the metabolic profile to identify wine characteristics, such as fermentation behaviours and antioxidant properties. Indeed, the analysis of metabolome was shown successful in identifying specific chemical compounds strictly related to the geographic production areas [115, 143]. The origin of wine could also be supported by the DNA-based analysis of must/wine microbiome [144–146]. Merging these three sources of data would result in a molecular label that is truly exhaustive and follows the Protected Designation of Origin (PDO) of wine.

Another application of metabolomics was on olive oil. Longobardi et al. [119] used a^1^H NMR fingerprinting combined with multivariate statistical analysis to authenticate extra virgin olive oils from seven different Mediterranean regions, demonstrating the possibility to predict the origin of olive oil samples with a very high confidence (>78%). At the DNA level, DNA barcoding cannot distinguish among different olive cultivars, whereas other genomics markers such as SSR and SNP were successful in achieving this goal [147]. DNA barcoding, combined with HRM (High Resolution Melting) analysis, was used instead to detect adulteration of olive oil with other oils [148]. Also in this case, genomics and metabolomics analyses could be complementary, to offer to the producer/consumer a comprehensive certification of origin and quality of oil.

An important aspect of food metabolome is that of flavour and aroma determination, which is often linked to the composition in volatile molecules. Dynamic headspace solid-phase microextraction (HS-SPME) followed by GC separation and high resolution MS analyser can be exploited to characterize the volatile components of some foodstuffs. With this approach, the volatile metabolomics pattern of beer raw materials has been defined in a recent paper [134]. Similar results were obtained with aromatic spices [149, 150] that have been also characterized using DNA barcoding approaches [44, 76]. In a strict sense, these results indicate that in the case of spices it is possible not only to identify the species but also the peculiar aromatic components responsible for their flavour and scent. Such combined analytical system can be seen as a way to also evaluate the efficacy of the processing of spices-based products along the entire supply chain (e.g., harvesting, exsiccation, grinding, and packaging).

Taking advantage of all these features and tools, NMR and MS are today able to answer most issues related to food analysis: (i) food traceability, authenticity, and safety, (ii) food composition and physical characteristics, (iii) food processing and storage, and (iv) food and health.

Thus, the study of the whole metabolic profile of food products can help defining quality features that make certain foods unique and can bring information on food safety and authenticity. For example, genetic modification, microorganisms colonization, and other food characteristics of major concern for human health are likely to influence large portions of the raw material or processed food molecular profile.

Another advantage of including the characteristics of the metabolome in the molecular label of a certain food is the potential of metabolomics in evaluating critical steps of the supply chain such as production, storage, and distribution. In 2014, Gallo and colleagues [116] described an interesting NMR application to study the influence of agronomical practices on the chemical composition of commercial table grapes. Specifically, the variability of the grape metabolome composition was evaluated considering primary metabolites, the compounds directly involved in the growth, and development of fruits. The authors found glucose, fructose, arginine, and ethanol as compounds quantitatively influenced by farming practices. Moreover, the comparison between organic and conventional productions showed a higher sugar content for the latter, resulting in a higher sugar-to-acid ratio [116].

In such a context, a metabolomics approach is complementary to a DNA barcoding analysis in evaluating the production processes as well as in monitoring the occurrence of alterations and species substitutions cases. For example, in 2015, Cagliani et al. [118] published an interesting application of metabolomics to characterize saffron, a very expensive and PDO spice. By using a multivariate statistical analysis of NMR data, they identified reliable biomarkers, specifically picrocrocin and crocins that permit distinguishing Italian products from other commercial varieties, where these peculiar compounds are less abundant (or even absent) [118].

The availability of an analytical platform based on the combination of genomics and metabolomics tools will have great potential in terms of food safety. As underlined in the first chapter, since its introduction in the 90's, the DNA-based diagnostics has developed different strategies to detect food pathogenic organisms. A DNA barcoding approach, combined with the use of HTS technologies, could certainly provide great advantages in this field because it would permit obtaining a comprehensive vision of all the putative food-related pathogens. However, this integrative panel of data would not be completely exhaustive because some microorganisms could be dead or inactive or become pathogenic only when they release specific toxins or metabolites [151, 152]. In this context, a metabolomics analysis based on MS/NMR approaches could provide important information regarding the occurrence of these metabolites or other compounds of major concern (e.g., antibiotics and pesticides) in foodstuffs. A rapid and simple analytical method, able to identify 255 veterinary drug residues in raw milk, was developed by Zhan and coworkers [128]. Their method was based on a two-step precipitation and ultra performance liquid chromatography coupled with electrospray ionization and tandem Mass Spectrometry (UPLC–ESI–MS/MS). Malachová et al. [129] optimized and validated in 2014 a LC–MS/MS method for the detection of 295 fungal and bacterial metabolites in four different types of food matrices: apple puree for infants (high water content), hazelnuts (high fat content), maize (high starch and low fat content), and green pepper (difficult or unique matrix).

Finally, recent studies have shown the possibility to link the metabolic profiling and characterization of foodstuffs to the screening of food matrices, aiming at the identification of small molecules able to bind and modulate the activity of a target protein (often involved in the etiology of specific pathologies). Techniques such as Saturation Transfer Difference- (STD-) NMR [153–155] and trNOESY NMR experiments [156, 157] allowed the identification of natural ligands present in* Salvia sclareoides* [136] and green tea [137], able to recognize, bind, and modulate the activity of A*β* peptides (whose aggregation processes are considered among the main biochemical events leading to Alzheimer's disease).

In conclusion, the future of food analysis will necessarily be based on the exploitation of integrative approaches, including both genomics and metabolomics. If in the past this was not feasible because of the lack of expertise and technical limitations, the current technological advances offer high performances in terms of standardization and universality to investigate a wide panel of food items. The spread of omics platforms, able to simultaneously process different matrices with a multiapproach strategy [111], unified under the control of bioinformatics tools, is boosting this revolution.

## 4. From Omics to Foodomics

The use of omics platforms to assess important aspects of food items (i.e., contaminants and bioactive molecules) is essential to obtain an exhaustive characterization of food quality and safety or to assess the effect of food on human cells, tissues, and organs as well. The availability of such platforms responds to a general trend in food science about the linking between food and health [7]. Nowadays, food is more and more considered not only as a source of energy but also as an affordable way to prevent future diseases. In this scenario, human health should be considered as a dynamic position in a multidimensional space [158] that spans from growth to development to reproduction. Early nutritional events (i.e., since the embryonic state) and food imprinting can define the trajectories of development and contribute to the wellness or the insurgence of noncommunicable diseases such as allergy, diabetes, and obesity [159]. In the development and maintaining ages, a proper nutrition could offer the better cost effective way to prevent such noncommunicable diseases [160]. Furthermore, undernutrition and overweight are global problems. The “global nutrition report” of 2013 highlights how the world is off-track to meet the 2025 World Health Assembly targets for nutrition [161]. Apart from social and economical issues, from the scientific point of view, nutrition research can furnish the keys for defining the characteristics of a proper nutrition. Therefore, a new discipline known as “foodomics” has been defined to study the food and nutrition domains through the application of advanced omics technologies to improve consumer's well-being, health, and confidence [162, 163]. Thanks to foodomics, many issues related to food could be addressed such as the evaluation of the effects of certain bioactive food components on biochemical, molecular, and cellular mechanisms, or the identification of gene-based differences among individuals in response to a specific dietary pattern [164–166]. Foodomics tools could permit identifying molecular biomarkers strictly related to the genes involved in the early stages of a certain disease and to elucidate the effect of bioactive food constituents on crucial molecular pathways for preventing future diseases with an adequate diet [166–168]. For example, a foodomics analysis was used to evaluate the effect of dietary polyphenols against colon cancer [169]. Ibáñez and coworkers [169] tested the chemopreventive effect of polyphenols from rosemary on the total gene, protein, and metabolite expression in human HT29 colon cancer cells. The results obtained from each component of the omics platform (i.e., transcriptomics, proteomics, and metabolomics) were integrated to estimate which cellular pathways were activated in response to polyphenols. Data suggests that polyphenols bring about an induction of cell-cycle arrest, an increase of apoptosis, and an improvement of cellular antioxidant activity. The genes, proteins, and metabolites involved in these three processes were identified thanks to the multiparameter omics analysis. It is important to underline the fact that the induction of apoptosis is especially relevant in colon cancer, since the renewal of the colon epithelium via apoptosis is the way used by the organism to eliminate deteriorated cells that can mutate to carcinogenic. Therefore, a diet rich in polyphenols plays an important role in the prevention of colon cancer.

Foodomics is a powerful discipline to identify the adding value properties of food items, as well as to detect food-related toxins and allergens or to assess the effects of food on human metabolism by evaluating cell-response [170, 171]. The efficacy of omics in the food sector also meets the emerging needs related to personalized nutrition [172]. A number of recent studies underlined the enormous variability of individual response to the same diet or food components: it is well known that food ingredients have effects that are unique to each individual, as unique as is its own transcriptome, proteome, and metabolome [158]. The role of foodomics does not finish once a personalized diet has been identified. Indeed, an exhaustive evaluation of the factors altering the metabolic properties of food components should also be taken into account. These factors include production process, methods, and duration of conservation, interaction with other components, cooking procedures, digestion, and interaction with microbiome [173].

The advantages of foodomics are relevant not only for producers but also for consumers to encourage a healthy diet and to reduce educational, behavioural, and economic barriers to accessing wellness. In this context, recent smartphone “apps” are becoming a powerful tool to promote the consumption of high-quality foodstuffs and in particular the consumption of those food items able to prevent diseases [174–177]. Such informative tools (including online portals and dissemination web sites) can be useful for different stakeholders to translate a molecular label based on omics approaches in a more understandable language for the whole category of consumers. The molecular labelling that combines DNA barcoding and metabolomics data with the information of foodomics represents a precious source of data to meet consumer requirements. In this sense, smartphone apps represent a simple tool able to share and translate molecular data to the various stakeholders of the food supply chain.

## Figures and Tables

**Table 1:** Updated list of DNA barcoding case studies in the field of food science and principal stakeholders. Producers are interested in valuing their crops or breeds by molecular certification; distributors are mainly interested in the traceability and authentication of traded products; the interest of consumers is to avoid commercial frauds/species substitutions and have an assurance on food provenance.

Food category	Target analysis	Interested stakeholders			References
		Producer	Distributor	Consumer	
Plants	Identification of species and provenance of *Mangifera* species	X	X	X	[65]
	Traceability of* Lycium barbarum* (Goji)		X	X	[66]
	Authenticity analyses of berry species	X	X	X	[67]
	Molecular identification of pineapple cultivars	X			[68]
	Identification of cocoa (*Theobroma* spp.; *Malvaceae*) cultivars	X			[69]
	Identification of date cultivars	X	X		[70]
	Identification of *Capsicum *species		X	X	[71]
	Authentication of PDO Fava Santorini (*Lathyrus clymenum*)	X	X	X	[72]
	Identification of Mediterranean bean species			X	[73]
	Identification and authentication of some Lamiaceae species		X	X	[44]
	Identification of *Thymus *species			X	[74]
	Authentication of saffron		X	X	[75]
	Authentication of black pepper powder		X	X	[76]
	Identification of *Salvia *species	X	X	X	[77]
	Authentication of herbal teas	X	X	X	[78]
	Authentication of turmeric powder (Zingiberaceae)		X	X	[79]
	Identification of herbs in beverages			X	[80]
	Authentication of fruits in jams		X	X	[81]

Mushrooms	Mushrooms identification		X	X	[82, 83]

Honey	Characterization of monofloral or multiflower honey	X		X	[42, 61]

Fishes and seafood	Identification of commercial fish species		X	X	[84–86]
	Identification of processed fish products			X	[87–92]
	Labelling authentication of fish products		X	X	[47, 51, 93–96]
	Identification of poisonous seafood species		X	X	[97]
	Identification of crab meat products			X	[98, 99]
	Origin and Authentication of Hairtail Fish and Shrimp	X	X	X	[100]
	Identification of *Octopus* species			X	[101]

Meat	Labelling authentication of game meat species			X	[45, 102, 103]
	Identification of ground meat products		X	X	[104]
	Identification of bovid species	X	X	X	[105]

**Table 2:** Examples of NMR and MS application in the field of food science.

Scope	Food category	Aim of the analysis	Analytical tool	References
Food traceability, authenticity, and safety	Saffron (*Crocus sativus *L.)	Quality and geographical origin	NMR	[118]
	Orange	Geographical origin	UPLC-qTOF-MS	[117]
	Raw milk	Safety: drug residues and other contaminants	UPLC–ESI–MS/MS	[128]
	Apple, hazelnuts, maize, green pepper	Safety: fungal and bacterial metabolites	LC–MS/MS	[129]
	Buffalo's mozzarella	Quality and traceability	NMR	[130]
	Olive oil	Geographical origin	NMR	[119]
	Wheat and bread	Geographical and varietal origin	NMR and IRMS	[120]

Food composition and physical characteristics	Grape	Effects of agronomical practices on composition	NMR	[116]
	Pork meat	Fatty acid chain composition	NMR	[131]
	Onion	Metabolic profiling	NMR and HPLC-MS	[132]

Food processing and storage	Wine	Effects of fermentation and aging	NMR	[115]
	Tea	Processing (variety)	LC–DAD-MS	[133]
	Beer	Profiling of raw materials for beer production	HS-SPME-GC-MS	[134]
	Coffee	Roasting process	NMR	[135]

Food and health	*Salvia sclareoides *	Compounds against neurodegenerative disease	STD-NMR	[136]
	Green tea	Compounds against neurodegenerative disease	STD-NMR	[137]
	Litchi (*Litchi chinensis Sonn.*)	Identification of bioactive compounds	NMR and MS	[138]
